# Health-related quality of life and associated factors in patients with myocardial infarction after returning to work: a cross-sectional study

**DOI:** 10.1186/s12955-020-01447-4

**Published:** 2020-06-17

**Authors:** Ruofei Du, Panpan Wang, Lixia Ma, Leon M. Larcher, Tao Wang, Changying Chen

**Affiliations:** 1 The College of Nursing and Health of Zhengzhou University, Zhengzhou, 450001 China; 2 School of Statistics, Henan University of Economics and Law, Zhengzhou, 450046 China; 3 Centre for Comparative Genomics, Murdoch University, Perth, WA 6150 Australia; 4 Department of Quality control, The First Affiliated Hospital of Zhengzhou University, Zhengzhou, 450052 China

**Keywords:** Myocardial infarction, Return to work, Health-related quality of life, Factors, Nursing

## Abstract

**Background:**

Return to work following myocardial infarction (MI) represents an important indicator of recovery. However, MI can cause patients to feel pressure, loneliness and inferiority during work and even detachment from employment after returning to work, which may affect their quality of life. The aims of this study were to identify the influencing factors of Health-related quality of life (HRQoL) in patients with MI after returning to work and explore the correlations between these factors and HRQoL.

**Method:**

This was a cross-sectional study. All participants were recruited from tertiary hospitals in China from October 2017 to March 2018. The general data questionnaire, Short-Form Health Survey-8 (SF-8), Health Promoting Lifestyle ProfileII (HPLPII), Medical Coping Modes Questionnaire (MCMQ) and Social Supporting Rating Scale (SSRS) were used to assess 326 patients with myocardial infarction returned to work after discharge. Multiple linear regression analysis was performed to explore factors related to HRQoL in patients with MI after returning to work.

**Results:**

The sample consisted of 326 patients. The mean total score of quality of life was 28.03 ± 2.554. According to the multiple linear regression analysis, next factors were associated with better HRQoL: younger age (B = − 0.354, *P* = 0.039), higher income (B = 0.513, *P* = 0.000), less co-morbidity (B = − 0.440, *P* = 0.000), the longer time taken to return to work (B = 0.235, *P* = 0.003), fewer stents installed (B = − 0.359, *P* = 0.003), participation in cardiac rehabilitation (CR) (B = − 1.777, *P* = 0.000), complete CR (B = − 1.409, *P* = 0.000), better health behaviors such as more health responsibility (B = 0.172, *P* = 0.000) and exercise (B = 0.165, *P* = 0.000), better nutrition (B = 0.178, *P* = 0.000) and self-realization (B = 0.165, *P* = 0.000), stress response (B = 0.172, *P* = 0.000), more social support such as more objective support (B = 0.175, *P* = 0.000), subjective support (B = 0.167, *P* = 0.000) and better utilization of social support (B = 0.189, *P* = 0.028), positive copping strategies such as more coping (B = 0.133, *P* = 0.000) and less yield (B = − 0.165, *P* = 0.000).

**Conclusions:**

HRQoL of MI patients after returning to work is not satisfactory. Health behavior, coping strategies, social support are factors which can affect HRQoL. A comprehensive and targeted guide may be a way to improve HRQoL and to assist patients’ successful return to society.

## Background

Globally, it is estimated that 17.5 million people die annually from cardiovascular disease, among which coronary artery disease is responsible for 7.5 million deaths [1]. As reported, there are more than 750,000 patients with myocardial infarction in the United States [2] and approximately 2,500,000 in China [3] up to now. It is expected that by 2020 the total number of patients with MI worldwide will increase by 75 million [4]. At present, percutaneous coronary intervention (PCI) has been widely used in MI treatment, effectively alleviating MI related symptoms and shortening the recovery period [5]. PCI could improve the prognosis of patients and facilitate patients to return to society earlier, therefore maximizing their quality of life [6]. This is particularly important for young MI patients, the incidence of whom saw significant increase in recent years. Indeed, as the main component of social productivity, returning to work is the key to restore self-esteem and self-confidence [7].

We define returning to work as people who were employed before illness return to work without long-term absence [8]. Occupational rehabilitation and reintegration are the ultimate goals of Cardiac Rehabilitation (CR) which is an important secondary prevention strategy for cardiovascular diseases [9]. As well-acknowledged, effective occupational rehabilitation can not only reduce the economic burden on society, family and individuals [10], but also serve as an indicator of returning to normal life. Successful workplace rehabilitation is of critical importance for cost-effectiveness [11] within the medical field. All these factors make the quality of life an important outcome measure for patients with MI after returning to work. Surely, maintaining a good HRQoL after returning to work is crucial for patients to cope with work pressure [12] and to coordinate the conflict between work and treatment [13]. Taking this into account, to help MI patients return to society and strength long-term social functions, a successful and effective treatment of MI should not only focus on prolonging the lifetime, but also pay attention to the HRQoL of patients after returning to work [14].

The rate of returning to work after MI is quite high. As shown by a recent study, approximately 63 to 94% of patients in the United States chose to return to work after MI within 6 months [15]. However, as a traumatic event, MI could detrimentally affect patients’ HRQoL both physically and mentally [16], leading to poor workplace rehabilitation (i.e. delayed return to work, increased sick leave, absence or even termination or resignation) [17]. As observed, patients who returned to work after MI could continuously be affected by impaired heart function (i.e. angina), resulting in limited physical activity [18]. On the other hand, people returning to work after MI are more likely to be affected by mental disorders such as depression, anxiety and experience ongoing stress, loneliness and inferiority [19, 20]. In fact, only a few people returning to work after MI could continue their work normally.

The HRQoL of these patients could be challenged by various factors [14]. Some researchers have shown that HRQoL could be detrimentally affected by low self-efficacy, insufficient disease management and poor communication [21, 22]. In addition, it was observed that age and disease status played pivotal roles in the HRQoL [23, 24]. Even though several studies have been conducted on the HRQoL and work status of patients with MI after returning to work, further investigation of such a topic is still necessary. Firstly, the key factors contributed to the physical, psychological and social recovery of patients have not been explored, although physical and psychological issues (reasons for enhanced work pressure and social psychological barriers [25, 26]) have been recorded among patients who returned to work after PCI [27]; secondly, reasons underlie the decline in quality of life of the patient have not been investigated. Given the chronic nature of MI, identifying the key factors related to the quality of life of patients with MI after returning to work, especially manageable factors through guidance and intervention are imperative. For the first time, the current study systematically investigated the potential factors that contribute to or prevent the improvement of HRQoL of MI patients returning to work. The findings of this study are expected to provide a practical guide to develop multidisciplinary solutions and interventions to further improve the overall treatment outcomes of MI.

## Methods

### Design

This work employed a cross-sectional study to assess the HRQoL, lifestyle, coping strategies and social support of patient with MI returning to work after hospital discharge.

In this study, health behavior (Factor A) was defined as healthy lifestyle and adherence to treatments; coping strategies (Factor B) were defined as attitudes and methods to face problems and challenges; social supports (Factor C) were defined as supports from friends, colleagues and family; and Factor X was defined as HRQoL. Our hypotheses are: (1) patients who had better health behaviors (Factor A) tended to have better HRQoL; (2) patients who had more positive coping strategies (Factor B) had better HRQoL, while those who had more negative outlook (Factor B) had worse HRQoL; (3) patients who were better socially supported (Factor C) had better HRQoL.

The theoretical basis supporting this study is the Moos’s crisis and coping model [28]. The model regards chronic disease as a life crisis for patients, which can change individual original cognition, and fundamentally change their health behaviors and coping strategies. Meanwhile, in the process of coping with chronic disease, individuals are influenced by various factors, with each of them resulting in different health outcomes. The model provides possible variables associated with this research.

### Settings and participants

A sample of 326 patients who had returned to work (Returning to work was defined as a patient who currently had payed employment and did not have long sick leave or extended absence from work.) were recruited from three tertiary hospitals in Zhengzhou, China. Inclusion criteria for participation in the study were: MI diagnosed within 2 years, age between 18 and 60 years, treatment with concurrent thrombolysis or interventional therapy. Exclusion criteria were: inability to provide informed consent and complete questionnaires and those who had difficulty in communicating.

The sample size was calculated based on G power 3.1.1 version with the effect size of 0.25, a desired significance level of 0.05 and a power of 0.80. The estimated minimal sample size was 144. Among 500 patients with MI after returning to work, 389 of them meeting the inclusion criteria were contacted and 42 of them refused to participate, 21 patients did not complete all the questionnaires. As a result, 326 patients were included in this study.

### Procedure and data collection

Before data collection, the detailed study design was presented to the nurse managers of each wards for informed consent. The list of potential participants was obtained from follow-up information systems. The principal investigator (PI) explained the purpose and processes of this study to all potential participants and then asked if they were willing to participate. The PI also informed them that they had the right to withdraw at any time without giving any reason.

Data was collected from October 2017 to March 2018 by structured questionnaires. Participants were recruited through telephone during follow-up. The questionnaires were completed by telephone inquiry or internet through sending a link to their mobile phones. We obtained every participant’s demographic characteristics including age, levels of education, marital status and so on. We also collected disease-related characteristics of every participant.

### Measures

#### Demographic characteristics

Sociodemographic and clinical data were collected from participants using patients’ file and a self-designed questionnaire including age, gender, education level, occupation, as well as open questions such as “What do you most worry about after returning to work?” and “What changes have you experienced psychologically and emotionally since returning to work?”

#### Short-form health Survey-8 (SF-8)

The SF-8 questionnaire was used to assess patients’ quality of life [29]. It is a generic instrument measuring 8 aspects of health: general health, physical pain, social function, emotional role, vitality, physical role, physical function and mental health. Scores in each item range from 1 (worst) to 5 (best), scores of total items range from 8 to 40, scores are presented after standardization through linear transformations. The higher score means better health condition. The Cronbach’s α was 0.87 ~ 0.95, the test-retest reliability was 0.92, and the validity was 0.82.

#### Health-promoting lifestyle ProfileII (HPLPII)

HPLPII is a specific and multidimensional self-reported scale for evaluating health behavior [30]. It consists of 6 domains: health responsibility (9 items), nutrition (9 items), exercise (8 items), self-actualization (9 items), interpersonal relationship (9 items) and stress coping (8 items). Each item uses a 4-point scale to measure the level of agreement with each statement (1 = never, 2 = seldom, 3 = sometimes, 4 = always). A score is calculated for each domain, and the sum of scores corresponds to the level of overall health behavior, ranging from 52 to 208. Higher scores indicate better health behavior. The Cronbach’s α was 0.80 ~ 0.91, the reliability was 0.89, and the validity was 0.86.

#### Medical coping modes questionnaire (MCMQ)

MCMQ was used to measure the patients’ coping strategies. It consists of 20 questions divided into 3 dimensions: facing (8 items), avoiding (7 items) and yielding (5 items). The responses to each question were rated on a scale from 1 to 4 (1 = never, 2 = seldom, 3 = sometimes, 4 = always) [31]. The total score for each domain was calculated and analyzed, higher scores of facing indicated more positive coping strategies while higher scores of avoidance and yield indicated more negative coping strategies. The Cronbach’s α of the 3 dimensions were 0.89, 0.87 and 0.90 respectively. The reliability was 0.90, and the validity was 0.84.

#### Social supporting rating scale (SSRS)

Social support was assessed with the SSRS. It contains 10 items grouped into 3 dimensions which cover objective support (3 items), subjective support (4 items) and the access to social support (3 items). The total score is the sum of the scores of each item. Higher total scores illustrate higher level of social support [32]. The Cronbach’s α coefficient is 0.88 ~ 0.92. The reliability was 0.92, and the validity was 0.90.

### Statistical analysis

Data were analyzed by SPSS 21.0. Quantitative data were analyzed using descriptive statistics to calculate sample demographics and clinical variables. In the descriptive analyses, means and standard deviations were calculated for continuous data while frequency and percentages were computed for categorical variables.

After confirming the eligibility of the assumptions for linear regression, multivariate analysis was performed to explore factors independently related to HRQoL using multiple regression. Independent variables were determined either by selecting normally distributed continuous variables that were related to HRQoL in line with previous publications or based on theoretical reasons. Pearson correlation analysis was applied to analyze the correlation between patients’ health behaviors, social support, coping styles and HRQoL. For all tests, a *p*<0.05 was considered statistically significant.

## Results

### Demographics of the study sample

A total of 326 participants were included in the study with more men (69.3%) than women (30.7%). The ages of these patients ranged from 32 to 60 years. Other key characteristics of the sample were also included (Table 1).

**Table 1:** Sociodemographic and Clinical Variables (*n* = 326)

Variables	Frequency (%)
**Marital status**	
Married	283 (86.8)
Unmarried	43 (13.2)
**Education Level**	
Junior school and below	103 (31.6)
High school	147 (45.1)
College and above	76 (23.3)
**Occupation**	
Farmer	88 (27)
Worker	135 (41.4)
Technicians	40 (12.3)
Office staff	22 (6.7)
Self-employed	33 (10.1)
Others	8 (2.5)
**Medical insurance**	
Yes	319 (97.9)
No	7 (2.1)
**Work Intensity**	
Light	107 (32.8)
Medium	219 (67.2)
**Income (Yuan)**	
<1000/M	41 (12.6)
1001 ~ 3000/M	83 (25.4)
3001 ~ 5000/M	159 (48.8)
>5001/M	43 (13.2)
**Participate in cardiac rehabilitation**	
Yes	206 (63.2)
No	120 (36.8)
**Complete cardiac rehabilitation**	
Yes	128 (39.3)
No	198 (60.7)
**Number of stent**	
0	70 (21.5)
1	213 (65.3)
2	29 (8.9)
≥ 3	14 (4.3)
**Time returned to work**	
<3 M	71 (21.8)
3 ~ 6 M	104 (31.9)
6 M ~ 1Y	88 (27)
>1Y	63 (19.3)
**Smoke**	
Never	128 (39.3)
Quit	13 (4)
Light	75 (23)
Medium	68 (20.8)
Heavy	42 (12.9)
**Drink**	
Never	124 (38)
Quit	20 (6.1)
Light	96 (29)
Medium	49 (15)
Heavy	37 (11.4)
**Co-morbidity**	
0	60 (18.4)
1	175 (53.7)
2	76 (23.3)
≥ 3	15 (4.6)

### Quality of life of patients after returning to work

As demonstrated in Table 2, the mean scores of 8 items ranged between 2.22 and 4.09. The mean total score of HRQoL was 28.03, more than half of the surveyed patients suffered from poor HRQoL who reported low scores (less than 25). Emotional roles showed lowest score followed by mental health and social functions.

**Table 2:** Quality of Life Outcome scores (*n* = 326)

Items/score range	Average Score (`x ± s)	Rank
General health / (1~5)	3.49 ± 0.81	3
Body function / (1~5)	4.09 ± 0.29	1
Physical Role / (1~5)	3.61 ± 0.59	2
Body Pain / (1~5)	3.18 ± 0.88	5
Vitality / (1~5)	3.23 ± 0.75	4
Social function / (1~5)	2.48 ± 1.08	6
Emotional Role / (1~5)	2.22 ± 0.87	8
Mental Health / (1~5)	2.38 ± 0.88	7

### Health behaviors, social support, coping styles

As can be seen from the Table 3, the mean total score of health behavior was 107.25. Most patients demonstrated a good interpersonal relationship with others and they thought they had realized their own value. Notably, most patients showed deficiency in health responsibility. The mean total score of social support was 32.07. Among the 326 patients, less than half had consulted health providers after discharge, while the number of patients seeking social and government assistance was low (32%). Furthermore, the patients were insufficient at identifying and using social resources. Patients tended to choose negative coping strategies with avoidance or yield (Table 3).

**Table 3:** Scores of Health Behaviors, Social Support, Coping Styles (*n* = 326)

Items/score range	mean score (^**−**^x ± s)
Health Responsibility/ (9~36)	15.70 ± 3.54
Nutrition/ (9~36)	17.48 ± 4.01
Exercise/ (8~32)	16.18 ± 2.97
Self-realization/ (9~36)	19.75 ± 4.91
Interpersonal Relationship/ (9~36)	19.35 ± 5.37
Coping with Stress/ (8~32)	16.83 ± 3.29
Total Score of Health Behavior/(52 ~ 208)	107.25 ± 10.21
Objective Support/(1~)	8.24 ± 2.13
Subjective Support/ (4~16)	18.00 ± 3.31
Utilization of Social Support/ (3~12)	5.83 ± 1.32
Total Score of Social Support/(8 ~ b)	32.07 ± 4.21
Facing/ (8~32)	17.51 ± 3.37
Avoidance/ (7~28)	22.58 ± 2.98
Yield/ (5~20)	14.15 ± 3.10

### Correlations between health behaviors, social support, coping styles and HRQoL

Pearson correlations analysis of main variables indicated that HRQoL was positively correlated with: health behavior (*r* = 0.528, *P*<0.01), health responsibility (*r* = 0.221, *P*<0.01), nutrition (*r* = 0.337, *P*<0.01), exercise (*r* = 0.247, *P*<0.01), self-realization (*r* = 0.454, *P*<0.01), stress response (*r* = 0.306, *P*<0.01), social support (*r* = 0.283, *P*<0.01), objective support (*r* = 0.181, *P*<0.01), subjective support (*r* = 0.181, *P*<0.01) and the utilization of social support (*r* = 0.154, *P*<0.01). However, there was no significant correlation between interpersonal relationship and HRQoL (*P*>0.05). Meanwhile, although HRQoL was positively correlated with facing (*r* = 0.300, *P*<0.01), it displayed a negative relationship with the yield (*r* = − 0.184, *P*<0.01). No significant association was found between avoidance and HRQoL (*P*>0.05).

### Factors of patients’ quality of life

Multiple linear regression analysis was performed to examine HRQoL-related factors. All variables, including demographic characteristics, health behaviors, social support and coping strategies were entered by stepwise variable selection with the forward selection and backward elimination methods being combined to filter the independent variables. After adding an independent variable, if the contribution of one of the previously added independent variables to the model fell below significance, the variable was eliminated. The disordered variables enter the model at the same time after dummy quantization. The independent variable was coded as follows: age: 18 ~ 45y =1, 46 ~ 60y =2; marital status: married =1, unmarried =2; education level: junior school and below =1, high school =2, college and above =3; medical insurance: yes =1, no =2; occupation: farmer =1, worker =2, technician =3, office manager =4, freelancer =5; work intensity: mild =1, moderate =2, severe =3, extremely severe =4; income: <1000 = 1, 1000 ~ 3000 = 2, 3001 ~ 5000 = 3, >5001 = 4; co-morbidity: none =1, 1 = 2, 2 = 3, 3and more than 3 = 4; stent number: none =1, 1 = 2, 2 = 3, 3and more than 3 = 4; time returned to work: <3 M = 1, 3 M ~ 6 M =2, 6 M ~ 1Y =3, >1Y =4; participate in CR: yes =1, no =2; complete CR: yes =1, no =2; smoke: yes =1, no =2; drink: yes =1, no =2. Health behavior, social support scores and coping strategy scores were substituted with actual values. As shown in Table 4, next factors displayed significant influence on HRQoL, including age, income, co-morbidity, the time taken to return to work, number of stents installed, whether to participate in or complete CR, health behaviors, social support and copping strategies. In addition, patients with better health behaviors such as more health responsibility and exercise, better nutrition, self-realization and stress response; better social support such as more objective and subjective support, better use of social supports; positive coping strategies such as facing showed significantly higher HRQoL.

**Table 4:** Factors related to patients’ quality of life (*n* = 326)

Independent variable	B	SB	t	***P***
Constant	7.581	1.168	6.491	0.000
Age	− 0.370	− 0.071	−2.211	0.028
Marital Status	−0.167	0.134	−1.576	0.276
Level of Education	−0.189	0.143	−1.475	0.301
Medical insurance	−0.041	0.186	−0.224	0.879
Occupation			−1.181	0.275
Farmer	−0.018	−0.003	− 0.051	0.959
Worker	0.327	0.026	0.571	0.522
Technician	0.369	0.047	0.803	0.423
Office Manager	0.209	0.019	0.662	0.460
Free Lancer	0.240	0.031	0.529	0.597
Working Intensity	−0.043	0.236	−0.257	0.794
Income	0.496	0.168	5.285	0.000
Co-morbidity	−0.501	−0.150	−4.783	0.000
Stent Number	−0.432	0.138	−2.957	0.006
Time Taken to Return to Work	0.258	0.104	3.365	0.001
Participate in Cardiac Rehabilitation	−1.680	−0.237	−7.803	0.000
Complete Cardiac Rehabilitation	−1.629	−0.288	−6.395	0.000
Smoke	0.083	0.075	0.938	0.331
Drink	0.013	0.100	0.012	0.917
Health Responsibility	0.175	0.243	7.853	0.000
Nutrition	0.186	0.292	9.324	0.000
Exercise	0.188	0.039	6.003	0.000
Self -realization	0.163	0.314	9.969	0.000
Cope with Stress	0.180	0.232	7.507	0.000
Health Behavior	0.157	0.359	16.512	0.000
Objective support	0.189	0.052	4.728	0.000
Subjective support	0.177	0.039	7.011	0.000
Utilization of social support	0.158	0.074	2.412	0.035
Social Support	0.159	0.263	8.499	0.000
Coping	0.138	0.182	5.285	0.000
Avoidance	0.034	0.116	0.912	0.291
Yield	−0.175	0.039	−6.930	0.000

R = 0.843, R^2^ = 0.710, Adjusted R^2^ = 0.699, F = 63.993, *P* < 0.001

## Discussion

MI is a common condition associated with coping strategies, health behaviors, social support and poor quality of life. We showed that almost half of the patients in this study reported poor quality of life. Patients with negative coping methods, unhealthy lifestyle, and less access to effective social resources demonstrated worse quality of life. According to our study, factors positively associated with HRQoL included family income, time taken to return to work, participation in and completion of CR, health behaviors, social support, and facing coping. In contrast, age, co-morbidity, number of stents installed and yield coping demonstrated negative relation to HRQoL.

According to this study, after returning to work, the body function, physical role and general health of patients were well recovered due to efficient treatments and rehabilitation via alleviation of symptoms [11]. Also, patients may choose suitable work to adapt to their physical functions to keep safety [12]. However, social function, mental health and emotional role of these patients were unsatisfactory. This was mainly due to next factors. Firstly, the difficulties for patients to coordinate the relationship between treatments and work [33]. As the work performance of these patients generally could not reach the pre-MI state, some of them worried whether they could continue to enjoy equal workplace-related rights. Secondly, family members were more likely to overprotect patients so that they preferred to pay more attention on physical condition of patients than their mental and emotional disorders. As a result, some patients were not encouraged to return to work by their families so that their social needs were ignored which led to loneliness and depression. Finally, there still lacks concern and support for such patients in China, and there are no suitable policies, insurance and welfare systems implemented in the mental and social health areas [8]. All these factors contributed to the anxiety, feeling of inferiority, social withdrawal, or even depression of patients, and compromised their self-esteem [34]. At the same time, patients had to adapt to new lifestyles which may result in restricted social activities [35]. As shown in this study, around 70% patients reported that they feared another MI, 23% found that they were more likely to lose their temper and patience. The longer these situations lasted, the higher chance for them to require long-term leave or be absent from work.

The results of this work suggested that there was a negative correlation between HRQoL and the time taken to return to work, which meant a faster return to work led to a lower HRQOL. This result was previously observed by Jalil et al. [33]. The time to return to work determines whether the patient has enough time to adjust and recover. Due to this fact, the earlier the return to work, the more impossible for patients to complete CR. In a short-term, such patients may find it difficult to manage disease or form good health behaviors, and find themselves in an insufficient situation to cope with adverse consequences. A study followed up patients 3 years after MI indicated that patients who had a harmonious relationship with disease were more likely to identify and evaluate risk factors [36]. It seems to take a relatively long time before potential risk factors becoming under control and patients becoming capable of managing their health. However, for economic reasons, some patients had to return to work as soon as possible with inefficient heart function and body function restoration which led to worse HRQoL. We suggest particular attention should be given to this population by both family and society.

According to a previous study, CR could improve cardiac function and activity tolerance of patients which contributed to better prognosis and HRQoL [37]. In this study, the HRQoL of the patients who participated in CR was significantly higher than those who did not. Our analysis suggested that through individualized exercise prescription, health guidance and behavioral intervention, CR can improve patients’ body function, assist in physical strength recovery, control risk factors and reduce negative emotions which affect mental and social adaptability. In this study, only 63.2% of patients participated in CR. The reasons for not participating included early return to work caused time and energetic restrictions, or distance (lived too far) from the rehabilitation center, etc. Moreover, the adherence of CR in this study needs to be improved. Less than 40% of patients completed CR, however, the completion of CR is a critical element to ensure the benefit of CR [38]. Most patients gave up the rehabilitation procedure because the benefits of CR did not seem obvious in the short-term. Hence the development of community-based or home-based CR programs is necessary, CR led by multidisciplinary team may be beneficial in this aspect. Besides, medical staffs should emphasize the long-term benefits of CR and arrange the projects reasonably.

It turned out that unhealthy lifestyle and behavior have a negative impact on HRQoL. A long-term cohort study of 69 patients with MI found that patients with better health behavior and displaying better compliance with treatment generally maintained higher HRQoL score [39]. In addition, health behaviors reflected individual attitudes towards decision-making. Patients with good health behaviors have more confidence to overcome difficulties and are more open to learn new knowledge and implement suitable methods for disease management. The first step to maintain good health behavior is to advise patients to take responsibility for their own health. Tangri et al. indicated that the health responsibility of patients depends on their awareness of health responsibility [35]. Our results also suggested that significant differences in health behaviors emerged despondent on patients’ attitude towards health responsibility. According to our study, most patients did not know what health responsibilities meant, and some even had never taken this issue into account. It was also found that patients were reluctant to participate in exercise. It was not only because they did not know what exercises suited their health conditions, but also the lack of knowledge about how to ensure safety during doing exercise. This indicated that a gradual health behavior plan assisted by health providers without extra burden should be put into practice. It is equally important to provide appropriate exercise information by professional physiotherapists before patients are discharged from the hospital.

HRQoL is also affected by the lack of social support or ineffective resource control. Adamczyk et al. [40] investigated 551 patients undergoing a CR program. Among them, 274 reported a low level of social support accompanied by a significantly lower HRQoL score. Social support affects the style and attitude of how patients cope with and manage diseases. In addition, Mollon et al. [41] showed that cardiac patients who couldn’t access to sufficient social resources were more likely to suffer a worse HRQoL. The family supports can make patients seek help more actively, therefore, enable them to acquire more methods/strategies to solve difficulties. In this study, we identified that patients with deficient social support had worse HRQoL, especially in some domains such as social function and emotional role. The emotional role is a subjective measure or the limitations of work or regular daily activities, while the social function is an assessment of physical and emotional problems in normal social activities. At the same time, we found that care, support and affirmation from family and society were vital for patients. In sight of this, it is imperative for health providers to help patients identify available social support and to break barriers to use social resources. More broadly, it is suggested that the colleagues and supervisors of patients treat them equally and arrange the workload reasonably according to their health conditions. Families and friends should not prevent patients from returning to work. Besides, it’s advocated that the whole society should pay more attention to these patients and develop supportive policies.

Another key factor related to HRQoL is coping strategies. Şahan and colleagues found that patients’ coping strategies could affect health outcomes and patients with positive coping strategies had better HRQoL [42]. In another case, Kureshi et al. [43] had investigated patients with coronary heart disease after returning to work and found that patients could not face the disease positively which lead to worse HRQoL. In most cases, patients were reluctant to supervise and evaluate health conditions, resulting in the failure to record and deal with symptoms in time. U Euler et al. [44] had confirmed that when a conflict occurred between disease and work, patients could not communicate well. Positive coping strategies can alleviate stress and promote communication, and have a significant effect through improving mental state and health behavior. This study also showed patients who yield easily got poorer HRQoL. This is understandable, yield weakened patients’ health responsibility, which may further lead to poor compliance with health behaviors. Moreover, if patients always yield and take an evasive attitude towards difficulties, their consciousness of disease prevention could be weakened, which leads to negative effect in managing disease. In turn, all these factors could eventually cause adverse cardiac events. Therefore, health providers should take targeted strategies to improve the patients’ coping strategies, enabling the patients to face disease and work with an optimistic and positive attitude.

### Advantages and limitations

To our knowledge, this is the first study that has investigated the effects of health behavior, coping strategies and social support on HRQoL of patients with MI after returning to work. However, it is necessary to highlight some limitations. Some participants rejected to attend or did not complete the whole investigation, which means that the results could be biased. The time constraints after returning to work probably contribute to this problem. Another reason could be that patients with very negative experience or attitude generally did not respond. At the same time, it should be noted that most patients in the study came from urban areas (cities); but according to the epidemiological characteristics of MI, the incidence in rural areas is higher. Since rural medical resources are limited and rural patients’ education level are generally low, future research should focus on these patients. Meanwhile, all the questionnaires used in this study are self-report questionnaires, while the measurement depended on the accuracy of patients’ answers. The interrelationship between the factors affecting HRQoL had not been explored, however, some variables may be moderator variables, which need to be further verified. One issue that can’t be ignored is that the sample was selected from three hospitals in one area without systematic sampling methods, which means the possibility of selection bias. Therefore, the results might be unsuitable to be extended directly.

### Implications

This study provided data on HRQoL of patients with MI after returning to work and pointed out key factors for improved HRQoL. Furthermore, the factors related to HRQoL were comprehensively analyzed to provide crucial information for health-care providers to not only identify and evaluate these factors, but also develop multidisciplinary interventions to improve HRQoL of MI patients returning back to work. The results of the study are important for promoting continuous care and thereby providing a foundation for in-depth understanding of direction of CR and factors that patients should pay attention to during physical social rehabilitation. On the other side, health-care providers should pay attention to the factors identified in this study and consider the following strategies: [1] providing individualized health education for patients and their relatives [2]; helping patients adhere to good health behaviors and improving their health responsibilities to further enhance activity endurance and maintain a balanced diet [3]; helping to appeal for support from the public and government agencies, to provide a good working environment and promote patients’ access to social support [4]; emphasizing and alleviating psychological and emotional disorders and promoting effective communication [5]; ensuring the safety of occupational rehabilitation training and carrying out integrated management [6]; guiding patients to adopt more positive coping styles and to keep optimistic attitudes.

## Conclusions

HRQoL of patients with MI who returned to work should be improved. Our findings suggested that health behavior, social support, and coping strategies were influencing factors on HRQoL of such patients. Good health behavior, sufficient social support and positive coping style are related to improved quality of life. Therefore, health providers should develop targeted and continuous interventions according to these factors in order to improve HRQoL and promote long-term social function and productivity. At the same time, the importance of vocational rehabilitation should be emphasized.

## Data Availability

The data generated during and/or analyzed during the current study are not publicly available, but are available from the corresponding author who was an organizer of the study.

## Abbreviations

MI: Myocardial Infarction
HRQoL: Health-related quality of life
CR: Cardiac Rehabilitation
SF-8: Short-Form Health Survey-8
HPLP II: Health-Promoting Lifestyle Profile II
MCMQ: Medical Coping Modes Questionnaire
SSRS: Social Supporting Rating Scale
